# Recycled Clay Brick Powder as a Dual-Function Additive: Mitigating the Alkali–Silica Reaction (ASR) and Enhancing Strength in Eco-Friendly Mortar with Hybrid Waste Glass and Clay Brick Aggregates

**DOI:** 10.3390/ma18122838

**Published:** 2025-06-16

**Authors:** Xue-Fei Chen, Xiu-Cheng Zhang, Ying Peng

**Affiliations:** 1 School of Civil Engineering, Putian University, Putian 351100, China; 2 Engineering Research Center of Disaster Prevention and Mitigation of Southeast Coastal Engineering Structures (JDGC03), Fujian Province University, Putian 351100, China

**Keywords:** construction and demolition waste, recycled clay brick, mortar

## Abstract

The construction industry’s escalating environmental footprint, coupled with the underutilization of construction waste streams, necessitates innovative approaches to sustainable material design. This study investigates the dual functionality of recycled clay brick powder (RCBP) as both a supplementary cementitious material (SCM) and an alkali–silica reaction (ASR) inhibitor in hybrid mortar systems incorporating recycled glass (RG) and recycled clay brick (RCB) aggregates. Leveraging the pozzolanic activity of RCBP’s residual aluminosilicate phases, the research quantifies its influence on mortar durability and mechanical performance under varying substitution scenarios. Experimental findings reveal a nonlinear relationship between RCBP dosage and mortar properties. A 30% cement replacement with RCBP yields a 28-day activity index of 96.95%, confirming significant pozzolanic contributions. Critically, RCBP substitution ≥20% effectively mitigates ASRs induced by RG aggregates, with optimal suppression observed at 25% replacement. This threshold aligns with microstructural analyses showing RCBP’s Al^3+^ ions preferentially reacting with alkali hydroxides to form non-expansive gels, reducing pore solution pH and silica dissolution rates. Mechanical characterization reveals trade-offs between workability and strength development. Increasing RCBP substitution decreases mortar consistency and fluidity, which is more pronounced in RG-RCBS blends due to glass aggregates’ smooth texture. Compressively, both SS-RCBS and RG-RCBS mortars exhibit strength reduction with higher RCBP content, yet all specimens show accelerated compressive strength gain relative to flexural strength over curing time. Notably, 28-day water absorption increases with RCBP substitution, correlating with microstructural porosity modifications. These findings position recycled construction wastes and glass as valuable resources in circular economy frameworks, offering municipalities a pathway to meet recycled content mandates without sacrificing structural integrity. The study underscores the importance of waste synergy in advancing sustainable mortar technology, with implications for net-zero building practices and industrial waste valorization.

## 1. Introduction

The construction industry, as one of the largest global consumers of natural resources and generators of industrial waste, faces mounting pressure to adopt sustainable material strategies aligned with circular economy principles [1]. Approximately 30% of construction and demolition waste comprises ceramic materials like clay bricks and glass, with current recycling rates below 20% in most regions creating both environmental burdens and untapped opportunities for resource recovery [2,3,4,5,6]. For example, Oasim et al. identified the feasibility of using recycled aggregate to generate new concrete [7]. Sandhu et al. used recycled concrete aggregate to replace partial natural coarse aggregate when preparing new concrete [8]. Vedrtnam et al. [9] used urban waste to improve the residual compression of concrete in post-fire exposure. Concurrently, the increasing incorporation of waste glass in cementitious materials—while addressing landfill diversion targets—introduces persistent challenges related to alkali–silica reaction (ASR), a deleterious chemical process causing destructive expansion and cracking in concrete structures [10,11,12]. Traditional ASR mitigation approaches, such as supplementary cementitious materials (SCMs) like fly ash [13,14,15] or slag, present logistical and availability constraints in the decarbonization era, necessitating alternative solutions derived from locally available waste streams. This dual crisis of material sustainability and durability forms the critical context for investigating recycled clay brick powder (RCBP) as a multifunctional additive in mortar systems containing hybrid waste glass and brick aggregates.

Recent studies have demonstrated the pozzolanic potential of calcined clay-based materials [16,17,18,19,20], with particular attention paid to their aluminum-rich composition influencing ASR suppression mechanisms. Unlike conventional SCMs, RCBP contains residual aluminosilicate phases and unreacted clay minerals that may simultaneously contribute to pore structure refinement and alkali-binding capacity [21,22]. When combined with waste glass aggregates—known for their high silica reactivity in alkaline environments—this system creates a complex interfacial zone where ASR kinetics and mechanical performance become interdependent. Previous research has primarily examined these materials in isolation: investigations into brick powder focus on cement replacement ratios while glass aggregate studies emphasize the effects of particle size on ASR [23,24,25]. However, the synergistic potential of combining these waste-derived components remains underexplored, particularly regarding how RCBP’s dual functionality (ASR inhibition and strength enhancement) manifests in systems containing both reactive glass and porous brick aggregates.

The chemical complexity arises from competing processes: waste glass aggregates release reactive silica susceptible to ASR [26,27] while brick aggregates introduce additional aluminosilicates that may alter reaction pathways. RCBP’s role extends beyond simple cement replacement as its particle morphology (angular microstructure with high surface area) and chemical composition (elevated Al_2_O_3_/SiO_2_ ratio compared to conventional SCMs) create unique interactions within this hybrid system. Laboratory evidence suggests that aluminum ions from RCBP may preferentially react with alkali hydroxides to form non-expansive aluminosilicate gels, thereby reducing the pH and silica dissolution rates critical for ASR propagation. Simultaneously, the filler effect of nano-sized brick powder particles enhances packing density at the aggregate–cement interface, a region typically weakened by the smooth surface and low absorption of glass aggregates. This dual mechanism proposal challenges the conventional wisdom that ASR suppression necessarily compromises early strength development, particularly in systems containing multiple recycled components with varying water demand and pozzolanic activity.

Current limitations in sustainable mortar design become apparent when examining life-cycle parameters as many “green” concrete formulations achieve environmental benefits at the expense of durability or require complex processing that negates their carbon advantage. The present approach innovates by utilizing two waste streams (construction waste and post-consumer glass) without high-energy pretreatment—recycled clay brick powder derives directly from crushing processes, and glass aggregates maintain their as-received morphology. Practical implementation considerations further underscore the research’s significance. Combining glass and brick aggregates creates a density-gradient material that may improve impact resistance while addressing the inconsistent supply of single-type recycled aggregates. Field data from pilot projects demonstrate that the rough surface texture of brick aggregates compensates for the poor bond strength typically associated with glass aggregates, although optimal blending ratios remain project specific. From a mixed design perspective, waste glass’s water-reducing effect counterbalances the increased demand from porous brick particles, enabling workability maintenance without superplasticizers—a crucial advantage for practical construction applications.

By establishing quantitative relationships between RCBP dosage, ASR suppression efficiency, and mechanical enhancement in hybrid aggregate systems, the research provides a template for designing multi-waste cementitious composites. The findings challenge the perception of construction waste as inferior materials, instead positioning them as active components in durability enhancement strategies. As regulatory frameworks increasingly mandate recycled content in public infrastructure projects, such dual-function systems offer municipalities a pathway to meet sustainability targets without compromising structural integrity—a paradigm shift essential for achieving net-zero ambitions in the built environment.

## 2. Experimental Details

### 2.1. Materials

**Figure 1:**
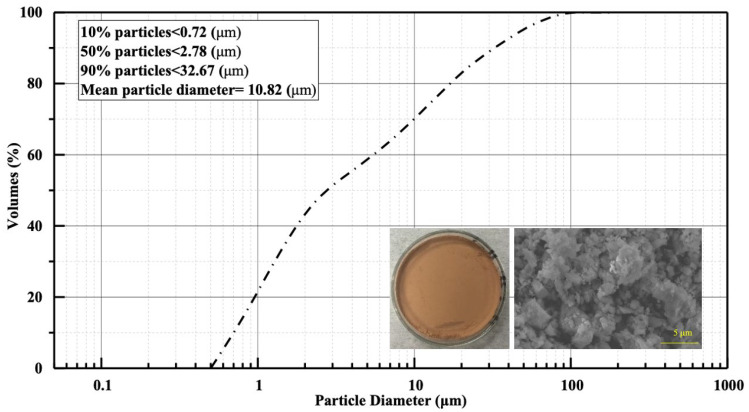
Particle size distribution and morphology of recycled clay brick powder.

The cement utilized was P.O 42.5 ordinary Portland cement procured from Hailuo Company (Wuhu, Anhui, China), with its primary chemical compositions and physical properties systematically documented in Table 1. The recycled clay brick powder (RCBP) was derived from recycled clay brick sand supplied by a local building material company, following the following preparation protocol: initial rinsing with tap water to remove impurities, subsequent drying in an oven at 105 °C for 48 h, and fine grinding using a Retsch PM400 planetary ball mill (provided by Verder (Shanghai, China) Instruments and Equipment Co., Ltd., Shanghai, China) at 400 revolutions per minute for 1 h. The ground material was then sieved through a 45 μm mesh to ensure 100% particle size distribution below 45 μm, followed by re-drying at 105 °C for 48 h prior to sealed storage. Figure 1 presents the visual characterization of RCBP, the particle size distribution, and the microstructural morphology, while the specific surface is determined as 0.895 m^2^/g.

**Figure 2:**
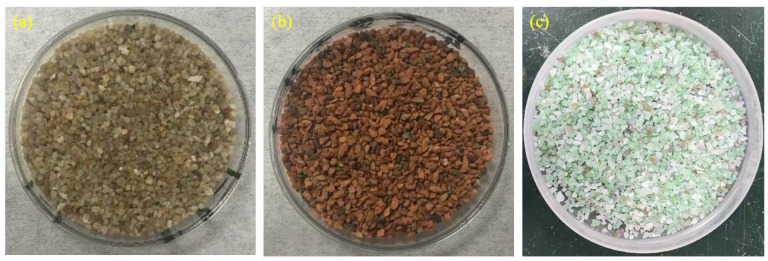
Morphology of aggregates: (**a**) standard sand; (**b**) recycled clay brick sands; and (**c**) recycled glass sands.

Aggregate materials comprised the following three distinct categories: Standard sand (SS) including both continuously graded and monosized (1.18–2.36 mm) fractions supplied by Xiamen ISO Standard Sand Co., Ltd. (Xiamen, China) in compliance with ISO specifications. The monosized fraction was obtained through screening using a vibrating sieve apparatus, producing the fine aggregate depicted in Figure 2a. Recycled clay brick sand (RCBS), exclusively in the 1.18–2.36 mm particle range, was sourced from the same supplier and macroscopically characterized in Figure 2b, with elemental composition provided in Table 2. Recycled glass sand (RG), also supplied by the local building material company, originated from waste flat glass and bottles with predominant transparent, green, and amber hues. This material was available in five particle size categories (150–300 μm, 300–600 μm, 600 μm-1.18 mm, 1.18–2.36 mm, and 2.36–4.75 mm), among which the 1.18–2.36 mm fraction was specifically utilized for fabricating mortar specimens. Visual examination of RG is presented in Figure 2c, with chemical composition analysis summarized in Table 2.

### 2.2. Sample Preparation

The mortar mix design for this experiment is detailed in Table 3, wherein the aggregates—recycled clay brick sand, recycled glass sand, and standard sand—were uniformly sized at 1.18–2.36 mm. The binder-to-sand ratio was maintained at 1:2.5, calculated based on the mass of oven-dried fine aggregates. For RCBS, which exhibits high water absorption, pre-saturation to achieve a saturated surface-dry (SSD) condition was performed prior to batching to ensure accurate water demand estimation. The binder matrix comprised 75 wt.% ordinary Portland cement and 25 wt.% recycled clay brick powder, with a water-to-binder ratio (*w*/*b*) of 0.5:1. The experimental mortar series were categorized into two primary groups: (1) RCBS was incorporated as a replacement for standard sand at substitution levels of 0, 20, 40, 60, 80, and 100 wt.%, and (2) RGS was used to replace RCBS in the mixes at identical substitution rates (0–100 wt.% in increments of 20 wt.%). Following casting, all mortar specimens were cured in a standard curing chamber maintained at 20 ± 2 °C and ≥95% relative humidity (RH) for 24 h prior to demolding. Subsequently, the specimens were transferred to a secondary curing environment set at 20 ± 2 °C and (60 ± 5)% RH until reaching the designated testing ages.

### 2.3. Testing

#### 2.3.1. Consistency and Fluidity

**Figure 3:**
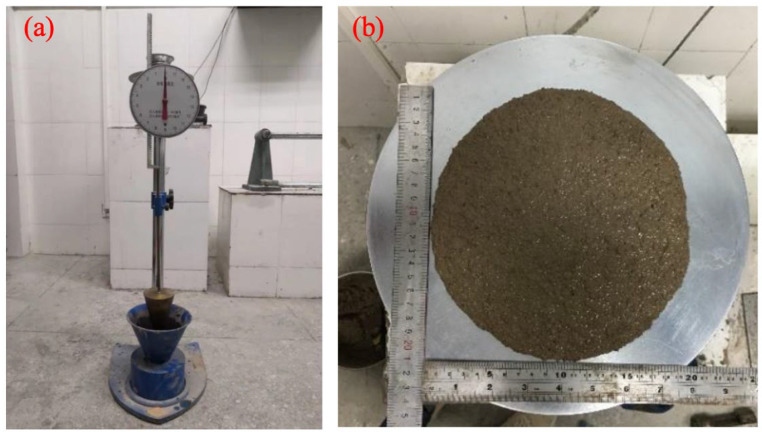
On-site test of consistency and fluidity: (**a**) consistency and (**b**) fluidity.

The mortar consistency was evaluated using a standardized mortar consistency tester (as illustrated in Figure 3a) in strict compliance with the JGJ70 specification. The experimental protocol adhered to the following systematic procedure: Initially, the surface of the test cone and inner walls of the container were moistened with a damp cloth. The mortar mixture was then carefully filled into the container to a level 10 mm below the brim, followed by 25 uniform insertions of the tamping rod from the center outwards. After this, the external walls of the container were gently tapped five times with the tamping rod to ensure proper compaction. The container was subsequently positioned on the base, and the cone tip was precisely adjusted to contact the mortar surface. The gauge pointer was calibrated to the zero position prior to measurement. Upon releasing the brake screw, the cone was allowed to penetrate the mortar under its own weight for exactly 10 s, after which the brake was re-engaged and the penetration depth was immediately recorded. Each sample was measured only once, and the final consistency value was determined as the average of three independent parallel measurements, ensuring the difference between replicates did not exceed 10 mm.

The mortar fluidity was evaluated per the GB/T2419, employing a specialized fluidity tester as shown in Figure 3b. The experimental procedure commenced with the thorough cleaning and moistening of the test mold, compaction rod, and table surface using damp cloths, followed by coverage with additional damp cloths to maintain optimal humidity conditions. The uniformly mixed mortar was then loaded into the mold in two distinct stages. The initial fill reached approximately two-thirds of the mold’s height and was subsequently compacted by manually tamping the mortar from the periphery towards the center 15 times. In the second stage, the mortar was filled to a level 20 mm above the mold’s brim and similarly compacted with 10 tamping motions. Upon removal of the mold casing, excess mortar was carefully leveled off and the truncated conical mold was detached. The table was then activated to execute 25 controlled jumps, facilitating the mortar’s fluidic movement. Post-jump, the fluidity was quantitatively assessed by measuring the diameter of the mortar spread along two perpendicular axes using a calibrated ruler. The final fluidity value was determined as the arithmetic mean of these three measurements, ensuring compliance with standardized testing protocols and enhancing the precision of the experimental outcome.

#### 2.3.2. Mechanical Properties and Water Absorption

**Figure 4:**
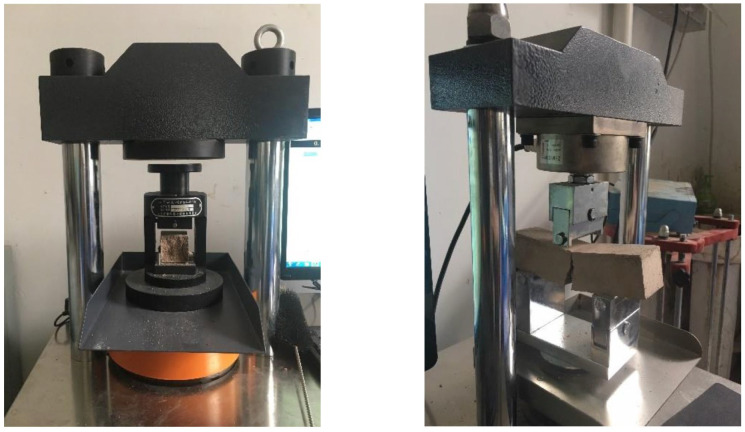
On-site graphs of mechanical strength testing: compressive strength (**left**); flexural strength (**right**).

The mechanical performance evaluation encompassed both flexural and compressive strength testing, executed utilizing specialized testing apparatuses as depicted in Figure 4. Prismatic specimens measuring 40 × 40 × 160 mm^3^ were fabricated in triple molds following the standardized curing protocols outlined in GB/T1767. These specimens were subjected to controlled environmental conditions within a standard curing chamber maintained at 20 ± 2 °C and ≥95% relative humidity (RH). The flexural strength was determined at 7, 14, and 28 days post-casting, employing a loading rate of 20 N/s with effective dimensions of 40 × 40 × 160 mm^3^. Three replicate specimens were tested, and their average value was recorded as the representative flexural strength for each batch. Subsequent compressive strength testing was conducted on fractured segments obtained from the flexural tests, utilizing a loading rate of 2.4 kN/s with effective dimensions of 40 × 40 × 40 mm^3^. Six replicate measurements were performed, and their average value was adopted as the compressive strength for each specimen group.

Concurrently, the water absorption of mortar was investigated following JGJ/T 70. Three prismatic specimens (40 × 40 × 160 mm^3^), cured under identical standard conditions (20 ± 2 °C, ≥95% RH) for 28 days, were oven-dried at 105 ± 5°C for 48 h before recording their initial mass (m_0_). These dried specimens were then fully immersed in water maintained at 20 ± 2 °C for 48 h, ensuring the formed surface faced downward. Post-immersion, surface moisture was removed using damp cloths, and the saturated mass (m_1_) was promptly measured. The water absorption coefficient (W_x_) was calculated as the average of three replicate specimens using Equation (1), which is as follows:

W_x_ = [(m_1_ – m_0_)/m_0_] × 100%. (1)

where W_x_ represents the water absorption percentage, m₁ the post-absorption mass in grams, and m_0_ the pre-absorption dry mass in grams.

#### 2.3.3. Micrographs via ESEM

The microstructural characterization was conducted using a Quanta FEG250 field-emission environmental scanning electron microscope (ESEM, FEI, Hillsboro, OR, USA), enabling high-resolution imaging of specimen surfaces. To prepare the samples for ESEM analysis, representative specimens of varying ages were precisely sectioned into 5 × 5 × 1 mm^3^ dimensions using a diamond blade. These segmented samples were then carefully stored in glass Petri dishes and subjected to a drying regimen in an oven maintained at 60 °C for 12 ± 0.5 h. Prior to microscopic examination, each dried sample was affixed to the ESEM specimen holder using conductive double-sided tape. A crucial preparatory step involved gold-coating the sample surfaces to enhance electrical conductivity, thereby facilitating high-magnification observation under the electron beam.

#### 2.3.4. Activity Index of Recycled Clay Brick Powder

The pozzolanic reactivity evaluation of supplementary cementitious materials (SCMs) is critical in assessing their efficacy as partial cement replacements as active components like SiO_2_ and Al_2_O_3_ undergo secondary reactions with hydration products (CH and high-alkalinity CSH) to generate low-alkalinity CSH, thereby enhancing the mechanical strength and durability of mortar or concrete matrices [28,29,30,31]. The pozzolanic activity index serves as a key indicator of SCM quality. Among various evaluation methods, the mortar strength ratio (i.e., activity index) method was selected for this study due to its quantitative precision and reduced susceptibility to operational biases compared to semi-quantitative techniques [31,32].

The mix design for the comparative strength method followed GB/T 12957 specifications, with detailed proportions outlined in Table 4. Continuously graded standard sand was employed as the fine aggregate, and prismatic specimens measuring 40 × 40 × 160 mm^3^ were cured under standard conditions (20 ± 2 °C, ≥95% RH) for 28 days prior to testing. The compressive strength ratio (K) was calculated as the ratio of the 28-day compressive strength of mortar containing 30% recycled clay brick powder (30RCBP) to that of the control mortar without RCBP (0RCBP). This ratio quantitatively reflects the pozzolanic activity contribution of RCBP, with higher K values indicating greater reactivity. The calculation was performed using Equation (2), which is as follows

K = (R/R₀) × 100%(2)

where K is the compressive strength ratio (%), R represents the 28-day compressive strength (MPa) of the RCBP-modified mortar, and R_0_ denotes the 28-day compressive strength (MPa) of the control mortar.

#### 2.3.5. Rapid Alkali–Silica Reaction (ASR)

The alkali–aggregate reaction (AAR) in concrete encompasses two primary reaction types, alkali–silica reaction (ASR) and alkali–carbonate reaction (ACR), with ASR being the most extensively researched phenomenon [34,35,36]. ASR involves a series of chemical interactions between alkaline components in concrete, such as NaOH and KOH, and reactive SiO_2_ present in aggregates. These reactions generate highly hygroscopic alkaline silicate gels that expand when they absorb water, inducing expansive stresses capable of causing concrete deterioration, including cracking and spalling. Mitigation strategies often involve incorporating pozzolanic supplementary cementitious materials like fly ash, metakaolin [37], or slag to consume excess alkalis and suppress ASRs [38]. The reaction mechanism of ASR is complex and has been simplified by some researchers [39,40] into four sequential steps which are represented by the following chemical Equations (3)–(6):

Na⁺ + OH^−^ + SiO_2_ → NaH·SiO_2_ (3)

Na⁺ + OH^−^ + NaH·SiO_2_ → Na_2_SiO_2_·xH_2_O (4)

NaH·SiO_2_ + SiO_2_ + H_2_O → Na_2_H_2_(SiO_2_)_2_·xH_2_O (5)

NaH·SiO_2_ + mSiO_2_ + H_2_O → Na_2_H_2_(SiO_2_)_(m+1)_·xH_2_O (6)

In contrast, the ACR occurs when aggregates containing limestone or dolomite react with concrete alkalis, leading to expansive deterioration. Unlike the ASR, the ACR does not produce cementitious reaction products at aggregate boundaries but rather involves the transformation of CaCO_3_ and Ca(OH)_2_. Notably, SCMs exhibit minimal effectiveness in mitigating ACR-induced expansion, distinguishing them from ASRs in terms of both reaction mechanisms and mitigation strategies.

**Figure 5:**
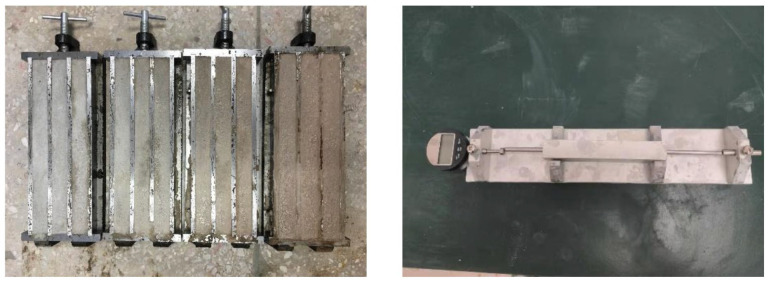
Rapid alkali–silica reaction test (ASR): steel molds (**left**); alkali–aggregate reaction comparator (**right**).

To investigate the potential and optimal dosage of recycled clay brick powder in mitigating the ASR induced by recycled glass sand, an accelerated mortar bar test (AMBT) was conducted in accordance with GB/T 14684. Seven replacement levels of cement by RBP (0, 5, 10, 15, 20, 25, and 30 wt.%) were evaluated to assess their effectiveness in ASR suppression. The AMBT procedure involved the following critical steps: Initially, mortar specimens measuring 25 × 25 × 280 mm^3^ were fabricated using steel triple molds (left subgraph of Figure 5). After casting, the molds were stored in a curing chamber maintained at 20 ± 2 °C and ≥95% relative humidity (RH) for 24 ± 2 h. The specimens were then carefully demolded, labeled, and placed in polypropylene containers filled with deionized water for complete immersion. These containers were sealed and transferred to an oven set at 80 ± 2 °C for an additional 24 ± 2 h of curing.

The initial length (l_0_) of each specimen was measured using an alkali–aggregate reaction comparator (right subgraph of Figure 5) with a precision of 0.001 mm. To minimize temperature-induced measurement errors, each specimen was removed from the oven, swiftly wiped with lint-free paper, and measured within 15 s. The measurement orientation and position were strictly maintained for all specimens. Subsequently, a 1 mol/L NaOH solution was prepared using analytical-grade NaOH and deionized water. The specimens, after initial length recording, were immersed in this solution within the polypropylene containers, ensuring complete submersion. The sealed containers were returned to the 80 ± 2 °C oven for continued curing. Length measurements were repeated at 3, 7, 10, and 14 days following the same protocol. The expansion rate for each specimen was calculated as the average of three replicate measurements using the following Equation (7):

ε_i_ = [(l_i_ – l_0_)/(l_0_ − 2Δ)] × 100% (7)

where εi represents the expansion rate at day i, l_0_ the initial length, li the length at day i, and Δ the gauge length.

A binder-to-sand ratio (cement to recycled glass sand mass ratio) of 1:2.25 and a water-to-binder ratio of 0.47:1 were adopted. Each test group, comprising three mortar specimens, required 440 g of binder and 990 g of recycled glass sand. Detailed proportions are provided in Table 5. The recycled glass sand was composed of the following five distinct particle size fractions: 150–300 μm, 300–600 μm, 600 μm–1.18 mm, 1.18–2.36 mm, and 2.36–4.75 mm. The mass of each size fraction was measured according to the distribution outlined in Table 6 to ensure precise control over particle size distribution in the mortar matrix.

## 3. Results and Discussion

### 3.1. Activity of Recycled Clay Brick Powder

**Figure 6:**
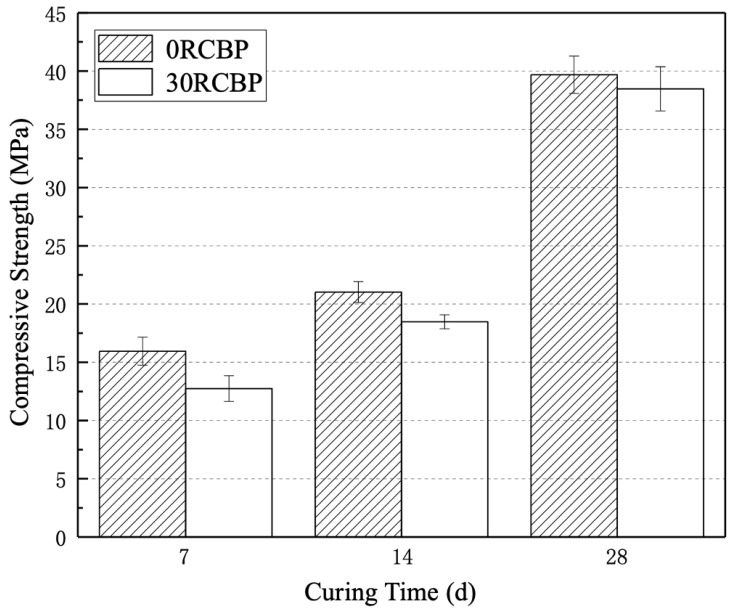
Compressive strength.

The pozzolanic activity of recycled clay brick powder (RCBP) is deemed qualified if the ratio K—defined as the 28-day compressive strength of mortar specimens with 30% RCBP substitution for cement divided by that of pure cement mortar specimens—meets or exceeds 65%. Higher K values indicate superior pozzolanic reactivity. In this study, RCBP particles (<45 μm) were blended with continuously graded standard sand to prepare mortar specimens following the pre-defined mix design and testing procedures. Figure 6 presents the mechanical performance comparison between the control group (0RCBP, pure cement mortar) and the experimental group (30RCBP, 30 wt.% cement replaced by RCBP).

**Figure 7:**
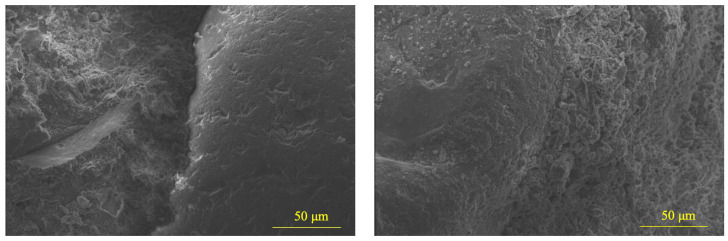
Micro-morphology of samples for the activity index test: 0RCBP-28d (**left**) and 30RCBP-28d (**right**).

Figure 6 illustrates the compressive strength development of mortar specimens incorporating 30 wt.% recycled clay brick powder (30RCBP) compared to the control group (0RCBP) across 7-, 14-, and 28-day curing periods. The 0RCBP specimens achieved compressive strengths of 15.95 MPa, 21.03 MPa, and 39.68 MPa at 7, 14, and 28 days, respectively, while the 30RCBP specimens exhibited values of 12.74 MPa, 18.48 MPa, and 38.47 MPa at corresponding ages. The pozzolanic activity index (K), defined as the strength ratio of 30RCBP to 0RCBP, increased progressively from 79.87% at 7 days to 87.87% at 14 days, reaching 96.95% by 28 days. This temporal enhancement aligns with findings by Afshinnia et al. [41], who reported comparable reactivity trends using 25% brick powder substitution. Notably, the 28-day K value substantially exceeds the 65% threshold, confirming superior pozzolanic performance. However, flexural strength measurements revealed a more pronounced sensitivity to RCBP substitution as 30RCBP showed a 24.66% reduction (5.5 MPa vs. 7.3 MPa for 0RCBP) at 28 days, which was contrasted by a 3.05% compressive strength decrease. The progressive increase in K values is attributed to synergistic mechanisms involving pore structure refinement and internal curing effects. Sub-45 μm RCBP particles act as microfillers, reducing interstitial voids between standard sand and cement. Meanwhile, their high water absorption (8.2%) enables gradual moisture release during hydration, sustaining reaction kinetics. SEM analysis at 1600× magnification (Figure 7) corroborates these mechanisms, showing denser interfacial transition zones in 0RCBP specimens compared to microvoid-rich 30RCBP matrices. Although RCBP’s water retention promotes late-stage strength gain, the resultant microvoids concentrate stress under flexural loading, explaining the disproportionate strength reduction. These findings validate RCBP as a high-potential supplementary cementitious material, achieving 96.95% cement-equivalent compressive performance at 30% substitution while enabling waste valorization and self-curing capabilities, albeit with trade-offs in flexural properties requiring consideration in structural applications.

### 3.2. Rapid Alkali–Silica Reaction

**Figure 8:**
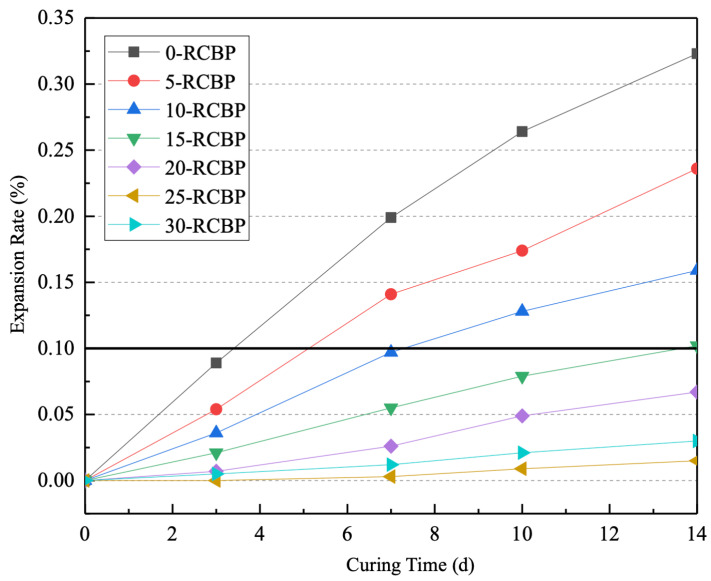
Development curve of the rapid alkali–silica reaction expansion rate.

Building upon the demonstrated pozzolanic reactivity of recycled clay brick powder from previous findings, this section herein investigates its potential to mitigate the alkali–silica reaction (ASR) induced by recycled glass sand. Given the inherent risk of ASR from the high silica content in glass aggregates, a systematic evaluation was conducted to determine RCBP’s suppression efficacy and optimal dosage. Following the accelerated ASR test protocol, mortar specimens containing 100% uniformly graded recycled glass sand as aggregate were prepared with cement replacement levels of 0%, 5%, 10%, 15%, 20%, 25%, and 30% RCBP by weight (designated as G0–G30). The experimental protocol involved monitoring length changes using an alkali–aggregate reaction comparator at 3, 7, 10, and 14 day intervals under standardized accelerated conditions (38 °C, 100% RH). Figure 8 presents the time-dependent expansion rates across seven experimental groups, revealing a distinct dosage–response relationship. Initial results demonstrate a critical threshold at 15% RCBP substitution, where expansion rates decrease precipitously from 0.18% (G0) to 0.07% (G15) at 14 days, followed by a plateau effect at higher substitutions. This nonlinear behavior suggests competing mechanisms: pore-solution alkalinity reduction through alkali binding at lower dosages versus dilution effects dominating at higher replacements. The measured expansion rates not only validate RCBP’s ASR suppression capability but also establish its dual functionality as both a pozzolan and ASR inhibitor in glass-incorporated cementitious systems.

The 14 day expansion rate (ε_14_) serves as the critical metric for assessing alkali–silica reaction risk under accelerated testing conditions. The evaluation protocol establishes three definitive thresholds. (1) Non-harmful classification (ε_14_ < 0.10%): Specimens demonstrating expansion below this threshold are conclusively deemed free from deleterious ASR potential. (2) High-risk classification (ε_14_ > 0.20%): Expansion exceeding 0.20% confirms significant ASR susceptibility, mandating preventive measures for structural applications. (3) Inconclusive range (0.10% ≤ ε_14_ ≤ 0.20%): Intermediate expansion values necessitate supplementary long-term testing (≥28 days) or microstructural analysis for definitive classification, reflecting the standard’s conservative approach to borderline cases. This tripartite evaluation framework enables rapid screening while acknowledging the complex kinetics of ASR development, particularly in systems containing reactive aggregates like recycled glass. The 0.10% safety margin accounts for measurement uncertainties (±0.02% per instrument calibration records) and material heterogeneity inherent in recycled components.

Figure 8 illustrates the influence of recycled clay brick powder substitution rates on alkali–silica reaction expansion kinetics across curing periods. The experimental data exhibit a coherent dosage–response pattern: specimens with 0–25% RCBP replacement demonstrate progressive ASR suppression as evidenced by monotonically decreasing expansion rates. Notably, 20% and 25% substitution groups achieved 14 day expansions (ε_14_) of 0.067% and 0.015%, respectively—both substantially below the 0.10% safety threshold specified in GB/T 14684. The 30% RCBP group maintained compliance (ε_14_ < 0.10%), confirming that ≥20% substitution effectively mitigates glass-induced ASR. Quantitative analysis reveals remarkable reduction efficacies of 79.26%, 95.36%, and 90.71% expansion decreases at 20%, 25%, and 30% substitutions relative to the control (0-RCBP). The existence of an optimal substitution level (25% RCBP) mirrors findings by Du et al. [42] regarding supplementary cementitious materials (SCMs) in ASR mitigation. Their comparative study established nonlinear relationships between SCM dosage and expansion suppression, identifying 5% silica fume and 20% fly ash as respective optima. This phenomenon arises from competing mechanisms. On the one hand, alkali-binding dominance below 25% RCBP is because the aluminosilicate phases sequester free Na⁺/K⁺ ions; whilst on the other hand, cement dilution effects beyond 25% are because reduced Ca(OH)_2_ availability limits pozzolanic reactions. The observed peak performance at 25% RCBP suggests an equilibrium between these counteractive processes, providing critical guidance for balancing ASR inhibition with mechanical performance retention in glass–aggregate mortars. This dosage optimization principle proves particularly crucial for applications where both durability and structural integrity are paramount.

**Figure 9:**
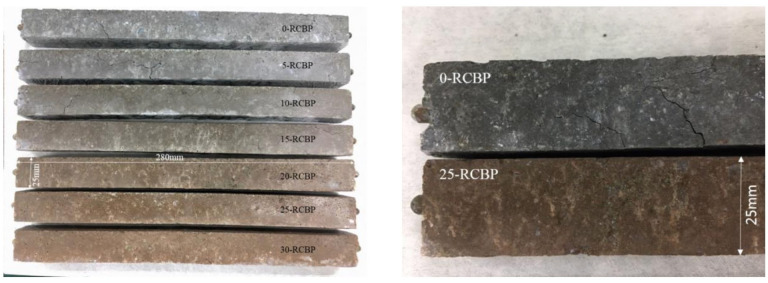
On-site crack morphology of samples suffering the 14 d rapid alkali–silica reaction test.

**Figure 10:**
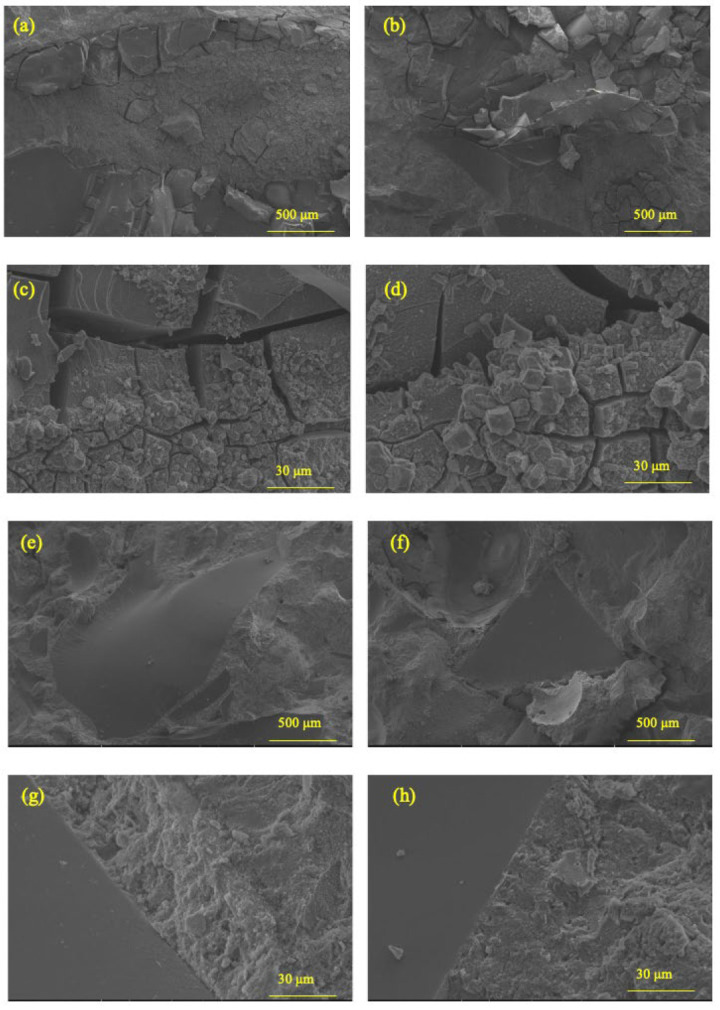
Micrographs of samples: (**a**–**d**) 0-RCBP; (**e**–**h**) 25-RCBP.

Figure 9 presents a comparative analysis of crack patterns observed in accelerated alkali–silica reaction mortar specimens after 14 days of curing. Specimens with 0%, 5%, and 10% RCBP substitution (denoted as 0-RCBP, 5-RCBP, and 10-RCBP) exhibited pronounced surface deformations accompanied by wide, interconnected cracks. In contrast, specimens containing 20%, 25%, and 30% RCBP (20-RCBP, 25-RCBP, and 30-RCBP) maintained dimensional stability with no visible deformations, although fine microcracks were detectable upon close inspection. These observations suggest a threshold RCBP content between 10% and 20% where microstructural integrity is significantly enhanced against ASR-induced damage. Microscopic examination of 0-RCBP specimens using scanning electron microscopy (Figure 10a–d) revealed severe fragmentation of recycled glass sand particles, forming disordered networks of microcracks at 200× magnification. Higher magnification (1600×, 3000×) highlighted secondary cracking within cement paste and poor interfacial bonding between glass sand and hydration products. Crystalline precipitates were observed on fractured glass surfaces, indicative of ongoing ASRs. Conversely, SEM analysis of 25-RCBP specimens (Figure 10e–h) demonstrated intact glass sand particles and dense cementitious matrix at 100× and 200× magnifications. At 3000×, both glass sand and binder phases exhibited crack-free surfaces with well-integrated interfacial zones, confirming effective mitigation of ASR by optimal RCBP incorporation.

**Figure 11:**
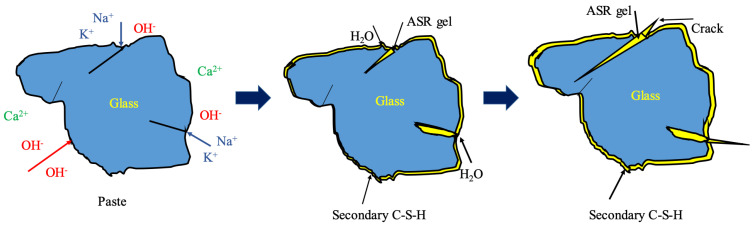
Schematic diagram of alkali–aggregate reaction caused by glass.

The schematic diagram in Figure 11 illustrates the proposed mechanism of ASR-induced damage in recycled glass sand by referring to previous studies [27,41]. Alkaline pore solutions react with reactive silica in glass particles, generating expansive gel products that create internal stresses. In under-substituted systems (≤10% RCBP) these stresses propagate unchecked, leading to macroscale cracking. However, sufficient RCBP content (≥20%) appears to consume excess alkali through pozzolanic reactions while its microfilter effect densifies the microstructure, thereby suppressing ASR and enhancing material durability. Previous studies [43,44,45] have elucidated the mechanisms through which pozzolanic admixtures suppress alkali–silica reactions. The findings demonstrate that (1) the incorporation of pozzolanic materials enhances the impermeability of cement paste while simultaneously reducing ionic mobility within the pore structure; (2) partial replacement of high-alkali cement with low-alkali pozzolanic admixtures effectively lowers the total alkali content of mortar, thereby reducing the alkalinity of pore solutions; (3) the addition of pozzolanic admixtures increases both the compressive strength of cement paste and its capacity to resist expansion stresses induced by ASR; and (4) reactive SiO_2_ present in pozzolanic admixtures chemically reacts with OH⁻ ions in pore solutions, promoting the formation of calcium silicate hydrate (C-S-H) phases that contribute to microstructure densification. These synergistic effects not only mitigate ASR-related damage but also improve the overall durability and mechanical performance of concrete systems.

### 3.3. Consistency and Fluidity

The aforementioned experimental findings demonstrated that recycled red brick powder not only exhibits significant pozzolanic activity but also effectively suppresses the alkali–silica reaction induced by recycled glass sand when substituted at levels above 20%. Notably, a 25% RRBP substitution rate was identified as optimal for mitigating ASR. Consequently, this substitution rate was adopted throughout the preparation of all mortar specimens in this study, serving as the basis for the 25% RRBP replacement ratio. The aggregates were prepared following the pre-defined mix proportions and operational procedures for the fabrication of mortar specimens. Specifically, these aggregates comprised standard sand, clay brick sand, and glass sand, each prepared with a uniform particle size distribution (1.18–2.36 mm). Given the relatively high water absorption capacity of recycled clay brick sand, it was necessary to pre-weigh the required amount of dry clay brick sand and subject it to saturated surface dry (SSD) conditioning prior to its incorporation into the mortar fabrication processes.

**Figure 12:**
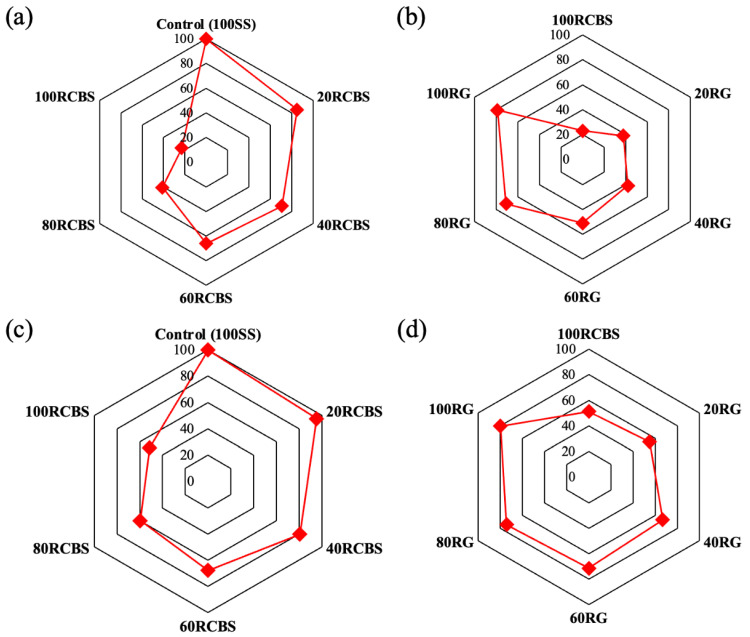
Consistency and fluidity relative to the control (sample mortar with 100% standard sand) (%): (**a**,**b**) consistency; (**c**,**d**) fluidity. Note: for (**a**,**c**) the numerical value indicates the substitution percentage of recycled clay brick sands relative to the control group, which is fabricated using 100% standard sands. For (**b**,**d**) the numerical value represents the substitution percentage of recycled glass sands in comparison to the mortar specimen composed entirely of recycled clay brick sands.

Figure 12a,b presents the specific values of consistency for mortars formulated with varying aggregate substitution ratios, encompassing 11 distinct groups categorized by aggregate type and substitution level. Clay brick sand was substituted for standard sand and glass sand at rates of 0 wt.%, 20 wt.%, 40 wt.%, 60 wt.%, 80 wt.%, and 100 wt.%. Notably, the 100RCBS mortar exhibited the lowest consistency value of 23 mm, while the 100SS mortar demonstrated the highest consistency at 100 mm. A discernible trend emerged where the consistency of mortars progressively decreased with increasing substitution ratios of clay brick sands for either standard sands or glass sands. Furthermore, when comparing mortars with equivalent clay brick sand substitution levels the SS-RCBS mortars consistently displayed higher consistency values than their RG-RCBS counterparts. Similarly, Figure 12c,d illustrates the fluidity measurements for these mortars. The 100RCBS mortar again registered the lowest fluidity at 122 mm, whereas the 100SS mortar achieved the highest fluidity of 238 mm. Analogous to the consistency trend, fluidity also diminished with elevated clay brick sands substitution rates and SS-RCBS mortars exhibited superior fluidity relative to RG-RCBS mortars at identical substitution levels.

Clay brick, a highly porous material formed through the high-temperature calcination of clay, inherently exhibits significant water absorption capacity. In this study, the recycled clay brick sand prepared via crushing processes demonstrates a water absorption rate of 17.1% and a bulk density of 1360 kg/m^3^. Recycled clay brick sands possess irregular shapes with rough surfaces lacking sharp edges, while concurrently displaying a substantial number of micropores. Notably, it exhibits the lowest bulk density among the three aggregates investigated. Under identical mortar-to-cement and water-to-cement ratios, increasing the substitution ratio of recycled clay brick sands in equal volumes of mortar results in a reduction in cementitious material per unit volume. Concurrently, the rough surface texture of recycled clay brick sands induces partial cement paste adherence within the pores, diminishing the effective cementitious matrix between aggregate particles. This phenomenon engenders heightened flow resistance during mortar movement, consequently manifesting as decreased fluidity and consistency in the mortar with elevated recycled clay brick sands substitution ratios.

Standard sand and recycled glass sand present bulk densities of 1570 kg/m^3^ and 1480 kg/m^3^, respectively, both demonstrating water absorption rates below 1%. RGS particles exhibit irregular shapes with smooth surfaces containing numerous sharp edges, coupled with dense, non-porous material properties. In contrast, SS particles approximate spherical geometry with smooth, compact surfaces devoid of both sharp edges and discernible fissures or pores. The intrinsic disparity in particle surface texture between glass sand and standard sand, particularly in terms of surface smoothness and angularity, contributes to differential rheological behaviors. Specifically, SS-RCBS mortar exhibits higher consistency and fluidity indices compared to RG-RCBS mortar under equivalent recycled brick sand substitution levels.

### 3.4. Mechanical Strength

**Figure 13:**
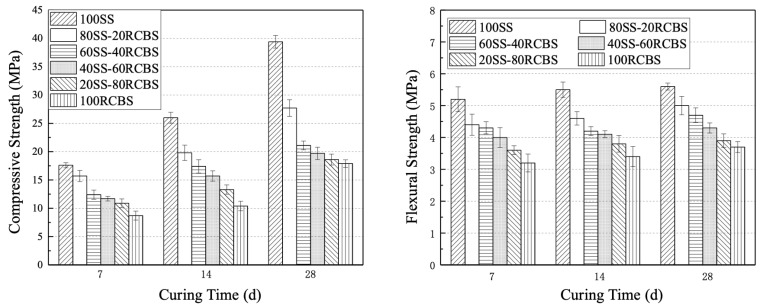
Compressive strength and flexural strength of mortar with various proportions of standard sands and recycled clay brick sands.

Figure 13 visually presents the compressive and flexural strengths of SS-RCBS mortar with varying substitution ratios of clay brick sand at 7, 14, and 28 days. The compressive strength of SS-RCBS mortar with different substitution ratios exhibits a distinct increasing trend with curing time. Notably, the 100SS mortar demonstrates the most significant growth rate among all SS-RCBS mortars, rising from 17.6 MPa at 7 days to 39.4 MPa at 28 days which represents a relative increase of 123.9%. As the substitution ratio of clay brick sand increases, the compressive strength of SS-RCBS mortar at all three ages decreases. The 100SS mortar exhibits the highest compressive strength at all ages, with values of 17.6 MPa, 26.0 MPa, and 39.4 MPa at 7, 14, and 28 days, respectively. Conversely, the 100RCBS mortar shows the lowest compressive strength at all equivalent curing times, with values of 8.7 MPa, 10.4 MPa, and 17.9 MPa at 7, 14, and 28 days, respectively.

Whilst the flexural strength of SS-RCBS mortar with varying substitution ratios also increases with age, the growth rate is significantly lower than that of compressive strength. The 100SS mortar exhibits the smallest growth rate in flexural strength among all SS-RCBS mortars, increasing from 5.2 MPa at 7 days to 5.6 MPa at 28 days which represents a relative increase of 7.7%. At the same substitution ratio of clay brick sand the flexural strength of SS-RCBS mortar at all three ages increases. The 100SS mortar demonstrates the highest flexural strength at all curing times, with values of 5.2 MPa, 5.5 MPa, and 5.6 MPa at 7, 14, and 28 days, respectively. The 100RCBS mortar, however, exhibits the lowest flexural strength at all equivalent curing times, with values of 3.2 MPa, 3.4 MPa, and 3.7 MPa at 7, 14, and 28 days, respectively.

The strength of mortar specimens is primarily determined by the hardness of the aggregate itself and the amount of cement filling between the aggregates after hardening. Standard sands have dense, smooth surfaces, approximate spherical shapes without edges, and higher bulk density and hardness than recycled clay brick sand. In contrast, recycled clay brick sand has a rough surface with numerous micropores and irregular particle shapes. Therefore, as the substitution ratio of clay brick sand increases under identical mix proportions, the content of cementitious material between aggregates in equal volumes of SS-RCBS mortar becomes relatively deficient, resulting in lower strength of hardened SS-RCBS mortar. Consequently, as the substitution ratio of clay brick sand increases, both the compressive and flexural strengths of SS-RCBS mortar decrease.

**Figure 14:**
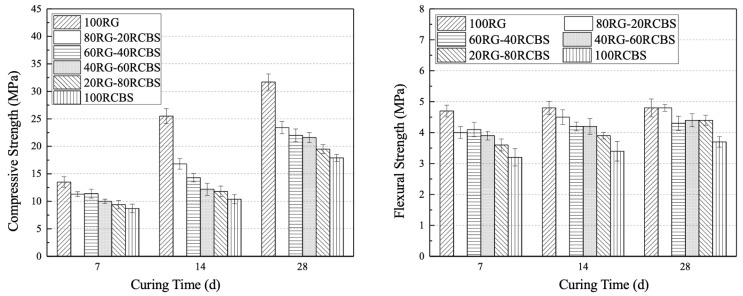
Compressive strength and flexural strength of mortar with various proportions of recycled glass sands and recycled clay brick sands.

Figure 14 visually presents the compressive and flexural strengths of RG-RCBS mortar with varying substitution ratios of clay brick sand at 7, 14, and 28 days. The compressive strength of RG-RCBS mortar with different substitution ratios demonstrates a clear increasing trend with age. Notably, the 100RG mortar exhibits the most significant growth rate among all RG-RCBS mortars, rising from 13.5 MPa at 7 days to 31.7 MPa at 28 days, representing a relative increase of 134.8%. As the substitution ratio of clay brick sand increases, the compressive strength of RG-RCBS mortar at all three ages decreases. The 100RG mortar demonstrates the highest compressive strength at all curing times, with values of 13.5 MPa, 25.5 MPa, and 31.7 MPa at 7, 14, and 28 days, respectively. Conversely, the 100RCBS mortar shows the lowest compressive strength at all equivalent curing times, with values of 8.7 MPa, 10.4 MPa, and 17.9 MPa at 7, 14, and 28 days, respectively.

While the flexural strength of RG-RCBS mortar with varying substitution ratios also increases with age, the growth rate is significantly lower than that of compressive strength. The 100RG mortar exhibits the smallest growth rate in flexural strength among all RG-RCBS mortars, increasing from 4.7 MPa at 7 days to 4.8 MPa at 28 days, representing a relative increase of 2.1%. As the substitution ratio of clay brick sand increases, the flexural strength of RG-RCBS mortar at all three ages decreases. The 100RG mortar demonstrates the highest flexural strength at all curing times, with values of 4.7 MPa, 4.8 MPa, and 4.8 MPa at 7, 14, and 28 days, respectively. The 100RCBS mortar, however, exhibits the lowest flexural strength at all equivalent curing times, with values of 3.2 MPa, 3.4 MPa, and 3.7 MPa at 7, 14, and 28 days, respectively.

The strength of mortar specimens is primarily determined by the hardness of the aggregate itself and the amount of cement filling between the aggregates after hardening. Recycled glass sand has dense, smooth surfaces but irregular shapes with distinct edges, and their bulk density and hardness are greater than those of recycled clay brick sand. In contrast, recycled clay brick sand has a rough surface with numerous micropores and irregular particle shapes. Therefore, as the substitution ratio of glass sand increases under identical mix proportions, the cement content between aggregates in equal volumes of RG-RCBS mortar becomes relatively abundant, resulting in greater strength of hardened RG-RCBS mortar. Additionally, the irregular shape of recycled glass sand enhances the mechanical interlocking between aggregates and between aggregates and cement. Consequently, as the substitution ratio of red brick sand increases, both the compressive and flexural strengths of RG-RCBS mortar decrease.

**Figure 15:**
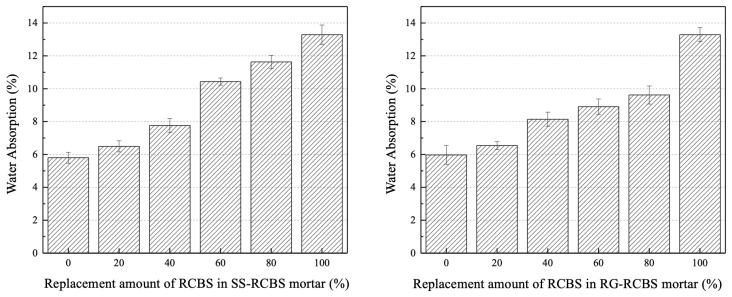
Water absorption of mortar with various proportions: SS-RCBS group (**left**); RG-RCBS group (**right**).

Figure 15 visually presents the 28-day water absorption rates of SS-RCBS mortar specimens and RG-RCBS mortar specimens with varying substitution ratios of clay brick sand. For both the SS-RCBS and RG-RCBS mortar specimens the water absorption rate increases with the substitution ratio of clay brick sand. The 100SS and 100RG mortar specimens exhibit minimum water absorption rates of 5.8% and 5.97%, respectively, while the 100RCBS mortar specimen demonstrates a maximum water absorption rate of 13.29%. The 28-day water absorption rate of the mortar specimens is primarily determined by the water absorption rate of the aggregate. The water absorption rates of standard sand and recycled glass sand are both less than 1%, whereas the water absorption rate of recycled clay brick sand used in this study is 17.1%, which is significantly greater than that of standard sand and recycled glass sand. Therefore, as the substitution ratio of clay brick sand increases, the 28-day water absorption rates of both SS-RCBS and RG-RCBS mortars show an increasing trend. The water absorption rate of mortar specimens serves as an important indicator for assessing the durability of mortar. The porosity within the mortar can be indirectly reflected by the water absorption rate of mortar specimens. Generally, a lower water absorption rate of mortar specimens indicates a lower porosity, suggesting a denser mortar structure and greater mortar strength. This pattern is consistent with the relationship between the water absorption rate and the strength of mortar specimens.

## 4. Conclusions

In the end, experimental findings reveal the multifaceted influence of recycled clay brick powder and sand on the properties of mortar specimens.

- Firstly, as the curing time increases, the activity index of mortar specimens with a 30% substitution ratio of recycled clay brick powder demonstrates a gradual upward trend, reaching 96.95% at 28 days, which is indicative of significant pozzolanic activity. Secondly, a substitution ratio of recycled clay brick powder of no less than 20% effectively mitigates the alkali–silica reaction induced by recycled glass sand, with the optimal substitution ratio being 25%.
- Furthermore, the consistency and fluidity of mortar exhibit a decreasing trend with an increasing substitution ratio of clay brick sand. Notably, SS-RCBS mortar specimens exhibit higher consistency and fluidity than RG-RCBS mortar specimens at the same substitution ratio. However, both the compressive and flexural strengths of SS-RCBS and RG-RCBS mortars decline with increasing substitution ratio of clay brick sand. Additionally, the growth rate of compressive strength significantly surpasses that of flexural strength for all mortar specimens as the curing time extends.
- Lastly, the 28-day water absorption rates of both SS-RCBS and RG-RCBS mortars show an increasing trend with the substitution ratio of clay brick sand. These findings collectively underscore the intricate interplay between the substitution ratio of clay brick sand and the performance characteristics of mortar specimens, providing valuable insights into the optimization of mortar formulations for enhanced durability and functionality.

While the findings support RCBP’s viability, future work will focus on long-term durability data (e.g., 12-month aging under cyclic conditions) and evaluate environmental impacts (e.g., CO_2_ footprint).

## Figures and Tables

**Table 1:** Primary chemical compositions and physical properties of cement.

Notation (%)	Mass
Calcium oxide (CaO)	66.43
Silica (SiO_2_)	18.4
Ferric oxide (Fe_2_O_3_)	4.03
Alumina (Al_2_O_3_)	3.73
Sulphuric anhydride (SO_3_)	2.65
Magnesium oxide (MgO)	2.11
Sodium oxide (Na_2_O)	1.36
Potassium oxide (K_2_O)	0.43
LOI	0.86
Density (kg/m^3^)	3160

Note: LOI—loss of ignition.

**Table 2:** Chemical compositions of recycled clay brick sands and recycled glass sands.

Notation	SiO_2_ (%)	Fe_2_O_3_ (%)	Al_2_O_3_ (%)	CaO (%)	MgO (%)	K_2_O (%)	Na_2_O (%)	SO_3_ (%)	BD (kg/m^3^)	WA (%)
RCBS	66.52	5.45	14.2	6.06	2.35	2.09	0.64	0.75	1360	17.1
RG	69.74	0.33	2.47	11.8	1.03	0.39	10.95	-	1480	0

Note: RCBS—recycled clay brick sand, RG—recycled glass sands, BD—bulk density, WA—water absorption.

**Table 3:** Mix proportion of mortar (relative proportion with binder as 1).

Notation	Water	Binder		Functionalized Aggregates			Total Alkali Content (Na_2_Oeq) (%)
		Cement	RCBP	RCBS	SS	RGS	
100SS	0.5	0.75	0.25	0.0	2.5	0.0	0.126
80SS-20RCBS				0.5	2.0	0.0	0.252
60SS-40RCBS				1.0	1.5	0.0	0.504
40SS-60RCBS				1.5	1.0	0.0	0.756
20SS-80RCBS				2.0	0.5	0.0	1.008
100RCBS				2.5	0.0	0.0	1.260
20RG-80RCBS				2.0	0.0	0.5	2.408
40RG-60RCBS				1.5	0.0	1.0	3.557
60RG-40RCBS				1.0	0.0	1.5	4.706
80RG-20RCBS				0.5	0.0	2.0	5.855
100RG				0.0	0.0	2.5	7.004

Note: RCBP—recycled clay brick powder, RCBS—recycled clay brick sand, RGS—recycled glass sand. The total alkali content (Na_2_Oeq) is calculated by the following equation: Na_2_Oeq = Na_2_O + 0.658 × K_2_O.

**Table 4:** Mix proportions of mortars for the activity index test. Reprinted from Ref. [33].

Notation	Cement, g	RCBP, g	Standard Sand, g	Water, mL
0RCBP	450	-	1350	225
30RCBP	315	135	1350	225

**Table 5:** Mix proportions of mortars for the rapid alkali–silica reaction test.

Notation	RCBP (g)	Cement (g)	RG (g)	Water (g)
0-RCBP	-	440	990	206.8
5-RCBP	22	418	990	206.8
10-RCBP	44	396	990	206.8
15-RCBP	66	374	990	206.8
20-RCBP	88	352	990	206.8
25-RCBP	110	330	990	206.8
30-RCBP	132	308	990	206.8

**Table 6:** Particle size distribution of recycled glass sand in the mortar for ASR test.

Size	150–300 μm	300–600 μm	600 μm–1.18 mm	1.18–2.36 mm	2.36–4.75 mm
Mass/g	148.5	247.5	247.5	247.5	99

## Data Availability

The original contributions presented in this study are included in the article. Further inquiries can be directed to the corresponding author(s).
